# Kinetics and Mechanisms of γ′ Reprecipitation in a Ni-based Superalloy

**DOI:** 10.1038/srep28650

**Published:** 2016-06-24

**Authors:** F. Masoumi, D. Shahriari, M. Jahazi, J. Cormier, A. Devaux

**Affiliations:** 1 Department of Mechanical Engineering, École de Technologie Supérieure (ETS), H3C 1K3, Montreal, QC, Canada; 2 Institute Pprime, UPR CNRS 3346, Physics and Mechanics of Materials Department, ISAE- ENSMA, BP 40109, Futuroscope- Chasseneuil Cedex 86961, France; 3 Aubert & Duval, Site des Ancizes, BP1, 63770 Les Ancizes Cedex, France

## Abstract

The reprecipitation mechanisms and kinetics of γ′ particles during cooling from supersolvus and subsolvus temperatures were studied in AD730^TM^ Ni-based superalloy using Differential Thermal Analysis (DTA). The evolution in the morphology and distribution of reprecipitated γ′ particles was investigated using Field Emission Gun Scanning Electron Microscopy (FEG-SEM). Depending on the cooling rate, γ′ particles showed multi or monomodal distribution. The irregularity growth characteristics observed at lower cooling rates were analyzed in the context of Mullins and Sekerka theory, and allowed the determination of a critical size of γ′ particles above which morphological instability appears. Precipitation kinetics parameters were determined using a non-isothermal JMA model and DTA data. The Avrami exponent was determined to be in the 1.5–2.3 range, suggesting spherical or irregular growth. A methodology was developed to take into account the temperature dependence of the rate coefficient *k*(*T*) in the non-isothermal JMA equation. In that regard, a function for *k*(*T*) was developed. Based on the results obtained, reprecipitation kinetics models for low and high cooling rates are proposed to quantify and predict the volume fraction of reprecipitated γ′ particles during the cooling process.

AD730^TM^ is a newly developed Ni-based superalloy for turbine disk applications, with reported superior service properties around 700 °C when compared to Inconel 718 and several other alloys^1^. This alloy is a γ′ strengthened alloy produced by the cast and wrought processes. In manufacturing processes such as rolling, forging, machining, friction welding and repair processes of turbine disks, different zones of the component experience temperatures above or below the solvus temperature of γ′ particles. Therefore, dissolution and subsequent γ′ reprecipitation occur during these thermomechanical processes^2^. In this context, an evaluation of γ′ characteristics and precipitation kinetics after cooling is critical in order to develop optimum process parameters and reach optimal mechanical performance^3^.

The precipitate size distribution depends strongly on the cooling rate. It has previously been reported^4^,^5^,^6^,^7^,^8^ that γ′ particles reprecipitate in monomodal morphology at high cooling rates, while multimodal distribution is obtained for slow cooling^9^,^10^,^11^,^12^,^13^,^14^. The formation of multimodal γ′ has been associated with multiple bursts of γ′ at different temperatures^10^,^12^,^13^,^14^. At lower undercoolings, just below γ′ solvus, higher diffusivity levels result in the formation of a first population of γ′ particles; while higher undercoolings provide supersaturation and thermodynamic driving forces for the formation of other populations of γ′ precipitates^4^,^10^,^11^. Furthermore, it has been reported that γ′ reprecipitation can be suppressed at high cooling rates in alloys with low volume fractions of γ′ (<30%)^15^,^16^,^17^,^18^. For example, no γ′ reprecipitation was observed in the fusion zone of laser and electron beam or linear friction welded Waspaloy^19^,^20^. This suppression leads to a precipitate-free region adjacent to the interface and, consequently, a pronounced drop in hardness in these regions. However, the influence of process and material parameters, such as the cooling rate, cooling start temperature, γ′ volume fraction, etc., on the formation of monomodal or multimodal precipitation of γ′ is still not well documented. It should also be noted that most of the reported works on γ′ reprecipitation have been focused on cooling from supersolvus temperatures, while little data^21^,^22^ is available on cooling from subsolvus temperatures.

In addition to the precipitate size distribution, the γ′ morphology is also a function of the cooling rate. Based on Mullins and Sekerka^23^ theory, in a diffusion-controlled process, when a second phase grows in a supersaturated matrix, there is a potential for morphological instability. Several examples of γ′ morphological instability and dendrites are available in the literature^24^,^25^,^26^ but few studies^27^,^28^ have investigated the origin of this instability, and specifically, the role of the cooling rate on inducing such instability. In the present study, the effect of the cooling rate on the morphological instability of γ′ particles in recently developed AD730^TM^ Ni-based superalloy is quantified, and the possible origin of this instability is investigated.

Little quantitative data is available on the kinetics of γ′ reprecipitation reaction in superalloys. Most studies^29^,^30^,^31^,^32^,^33^,^34^ have been focused on the effect of different aging heat treatments on the size and morphology or coarsening kinetics of γ′ in superalloys. Rougier *et al*.^35^ developed a particle size distribution (PSD) model for numerical simulations of γ′ precipitation during isothermal aging of NiCrAl superalloys. They used a multicomponent diffusion model in order to calculate the growth rate. However, their model was fully coupled with CALPHAD (Computer Coupling of Phase Diagrams and Thermochemistry) for the calculation of the nucleation driving force, and this coupling is very expensive computationally. Y. Wang *et al*.^9^,^10^,^11^ used phase field modeling to visualize the microstructure development and quantify physical phenomena such as impingement, particle coalescence or splitting by solving nonlinear time-dependent phase field equations within the framework of irreversible thermodynamics. However, to obtain accurate results, particularly for a new alloy, extensive experimental work is needed to set realistic values for the boundary conditions and determine material parameters. Therefore, besides its valuable benefits, its application to new alloys is not straightforward.

Bonvalet *et al*.^36^ recently proposed a numerical model for γ′ precipitation during the isothermal heat treatment of NiCrAl alloys. The model is based on the particle coarsening theory of Philippe *et al*.^37^, and cannot be directly applied to continuous cooling processes and to transient cooling conditions such as the one used in the present investigation. Olsen *et al*.^38^,^39^,^40^ used the PrecipiCalc^TM^ software to develop models for PSD and transformation rates. The software is built around thermodynamic computations and multicomponent diffusional nucleation and growth models to simulate multiphase precipitation. However, since the operation of this software is based on CALPHAD databases, calibration and independent experimental measurements need to determine the model parameters with high fidelity and minimum overfitting for each new alloy. As a consequence, these methods are not easy to apply for industrial applications due to the large computational time required. Many physical constants, such as element diffusion coefficients, surface energies, interface kinetic coefficients and driving forces for phase transformations, are also needed for obtaining reliable results in numerical models; this data is not always readily available for alloys with complex compositions^41^. The situation becomes even more complicated when a new alloy, such as the one used in the present investigation, is considered.

In order to overcome the limitations of numerical methods, semi-analytical models calibrated by experiments may be used. The Johnson-Mehl-Avrami (JMA) model^42^ is one of the most important semi-analytical models available, and plays a central role in transformation studies, where nucleation and growth mechanisms operate. The model has been widely applied to γ′ volume fraction evolution during isothermal dissolution and aging treatments^33^,^43^,^44^. However, to the knowledge of the authors, no such model exists for the quantification of the kinetics of reprecipitation reaction and its mechanisms during the continuous cooling process of Ni-based superalloys. The JMA model holds some constraints in the case of isochronal transformations. Mittemeijer *et al*.^45^ extended the JMA equation to non-isothermal transformations. However, the applicability of the model to complex alloy systems, such as Ni-based alloys, has not been reported.

On the basis of the above analysis, the objectives of this study are:

1. To analyze the size distribution and morphology of multiple populations of γ′ precipitates formed during continuous and interrupted cooling from supersolvus and subsolvus temperatures.
2. To develop and validate a semi-analytical model to predict γ′ reprecipitation kinetics at low and high cooling rates.
3. To develop a better understanding of fundamental mechanisms governing γ′ reprecipitation and its morphological instability as a function of the cooling rate.

## Precipitation Kinetics

For the analysis of precipitation kinetics, a physical property such as specific volume/length or enthalpy of the material can be investigated as a function of time and temperature. Then, the fraction of transformation, *Y*, can be described by following JMA equation, which is applicable to both isothermal and non-isothermal analysis^42^,^45^:


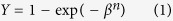


where *n* is the Avrami exponent which depends on precipitate growth modes. For non-isothermal transformations, *β* is written as:


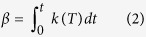


where *k*(*T*) = *k*_0_(*T*)*exp*(−*Q*/*RT*) is the Avrami rate parameter which depends on the nucleation and growth rate and varies with *t*. Analytical approximation of the above integral using a limited development as proposed by Mittemeijer *et al*.^45^ gives:


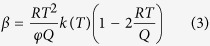


where *R* is the gas constant, *φ* the cooling rate and *Q* the activation energy of the reaction. Values of *t*, *T*, *Y* and the kinetics parameters, *n*, *Q* and *k*(*T*) representative of the precipitation process can be obtained from DTA measurements using the following procedure.

In a DTA run, the fraction of the precipitation *Y*(*T*) at temperature *T* is given by^46^,^47^:


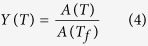


where *A*(*T*) is the area under the peak between the initial temperature of the peak *T*_*i*_ (i.e. temperature of precipitation onset) and temperature *T*, and *A*(*T*_*f*_) is the area of a peak between *T*_*i*_ and *T*_*f*_ in which *T*_*f*_ is the final temperature of the peak (i.e. temperature of precipitation end).


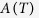
is defined as:


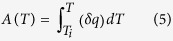


where 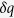 is obtained during a constant cooling rate test by subtracting the measured heat flow of the sample from that of the inert reference.

The precipitation kinetics, *dY*/*dt*, can be related to cooling rate, *φ*, in DTA scan by:


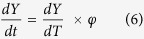


The growth exponent, 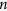, can be obtained from the transformed fraction, 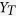, attained at a certain value of 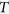, as measured for different cooling rates by^48^:


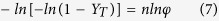


Finally, the activation energy (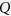) of the process during non-isothermal cooling can be determined by^45^:


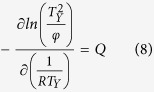


where *T*_*Y*_ is the temperature which is attained at a certain fixed value of *Y*. Using the above equations, it is possible to determine the kinetics of γ′ precipitation during continuous cooling and to study the governing mechanisms by determining 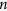, *k*(*T*) and *Q*.

## Experimental Procedure

The AD730^TM^ material was provided by the Aubert & Duval company in the form of a forged square bar. The bar had been solutionized at 1080 °C for four hours, and then air-cooled to room temperature. The chemical composition of the alloy is given in Table 1. The equilibrium volume fraction of γ′ is around 40%.

The DTA technique is a well-established technique for studying the kinetics of precipitation or phase changes in alloy systems^49^. The continuous cooling tests were carried out using a Diamond TG/DTA PerkinElmer with a Pt crucible and flowing Ar atmosphere. DTA experiments were conducted in order to determine γ′ transformation temperatures, to follow the evolution of the γ′ size, morphology and volume fraction during cooling, and to measure the heat flow. All experiments were conducted using high purity Ar to minimize potential oxidizing effects. The unit was calibrated using pure aluminum and gold, with precise melting points of 660 °C and 1064 °C, respectively. The samples were 2 mm × 2 mm × 0.5 mm in size, and were heated at rates of 10, 15, 65 and 120 °C/min, up to 1200 °C, held for one minute, and then cooled down to room temperature by the same rates of 10 °C/min (SC_10_: Slow Cooling), 15 °C/min (SC_15_), 65 °C/min (IC: Intermediate Cooling) and 120 °C/min (FC: Fast Cooling). The cooling rates were chosen in order to mimic the quenching of a reasonably large superalloy forged disc in air or oil. The 10 °C/min cooling rate is typical of the conditions encountered in industry for equiaxed solidification.

For accurate and reliable measurements of the heat flow, all the DTA runs were corrected by subtracting the measured heat flow of the sample from that of a DTA baseline obtained from a run with an empty pan. In the DTA plots, the endothermic and exothermic reactions were plotted downward and upward, respectively. In order to validate the equation predicting γ′ size evolution obtained from DTA continuous cooling experiments, samples were heated at a rate of 120 °C/min, up to 1200 °C, held for one minute, and then water-quenched (WQ) to room temperature using the Gleeble™ 3800 thermomechanical simulator. The microstructure of the samples, and particularly the γ′ characteristics, were then examined with FEG-SEM.

Discontinuous (interrupted) cooling tests were also carried out to study different nucleation bursts of γ′ during cooling cycles from subsolvus temperatures. For these tests, samples were heated from room temperature to 1100 °C at a rate of 120 °C/min, and held one minute at this temperature. The interrupted cooling consisted in continuous cooling at a constant rate of 120 °C/min, followed by immediate water quenching at 1040 °C or 780 °C.

For microstructure characterization, the specimen surface was prepared following standard metallographic preparation procedures and etched using a mixture of Regia water (2/3) and distilled water (1/3). Microstructural examination of the etched samples was carried out using a Hitachi SU70 FEG-SEM. The characterization of the nanometric size particles and morphology of the precipitates were conducted in secondary electron (SE) and back-scattered electron (BSE) modes. Analyses were carried out using small and high magnification SEM images ranging from 2000 to 100,000 magnifications in order to investigate the γ′ distributions and morphologies. In order to measure the dimensions and volume fractions of the γ′ precipitates, digitized microscopic images and ImageJ analysis software were used. Each reported value for γ′ size or volume fraction is an average of 5 measurements. In each case, area measurements on more than 100 precipitates were carried out using the ImageJ software. The particle radius was then calculated as the radius of a circle whose surface area equaled that of the corresponding particle.

## Results

### Continuous Cooling Precipitation from Supersolvous Temperature

#### DTA Data Analysis

**Figure 1:**
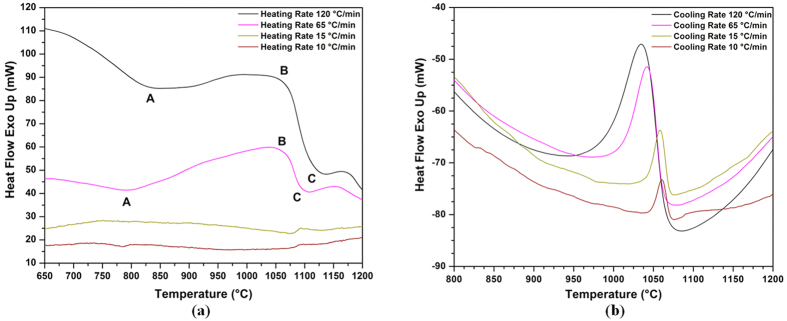
DTA curves showing (a) γ′ dissolution and precipitation peaks during heating for different heating rates (b) γ′ precipitation peaks during cooling for various cooling rates.

Figure 1 shows DTA diagrams with different endothermic and exothermic peaks showing the dissolution and precipitation in the AD730^TM^ alloy measured for the four investigated heating and cooling rates. Two endothermic peaks can clearly be observed at the 65 °C/min and 120 °C/min heating curves. Peak A occurs around 800 °C, and corresponds to the dissolution of secondary γ′ particles. Peak C is related to the dissolution of primary γ′ and is observed for all heating rates. This peak is around 1080 °C for the 10 °C/min heating rate and 1120 °C for the 120 °C/min heating rate.

Figure 1(b) shows the results of the cooling portion of the DTA diagram, and the phase transformation temperatures values obtained during cooling are provided in Table 2. It can be seen that the first burst of nucleation initiates at 1075 °C for the SC_10_ condition and at 1070 °C for the FC condition. Both bursts occur below Peak C (i.e., primary γ′ solvus temperature). As the cooling rate increases, the peak shifts to lower temperatures and undercooling increases. For example, the peak temperature is around 1060 °C for the SC_10_ sample, while it drops to 1035 °C for the FC condition.

**Figure 2:**
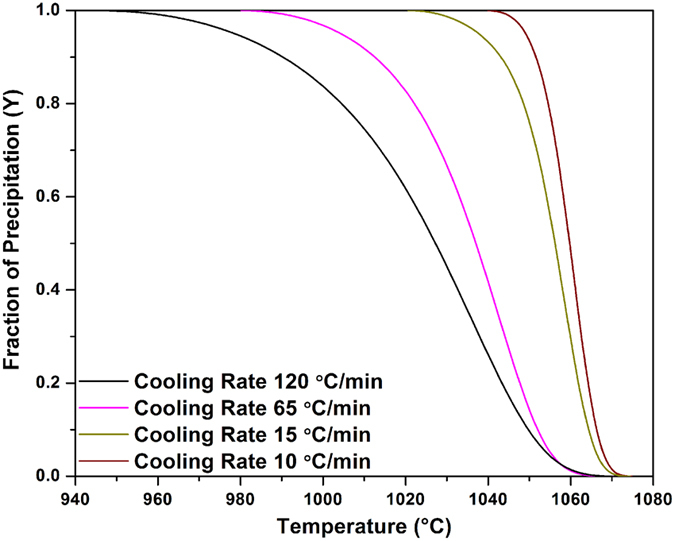
Amount of γ′ precipitation as a function of temperature for various cooling rates showing the curves shift to lower temperatures with increasing the cooling rate.

**Figure 3:**
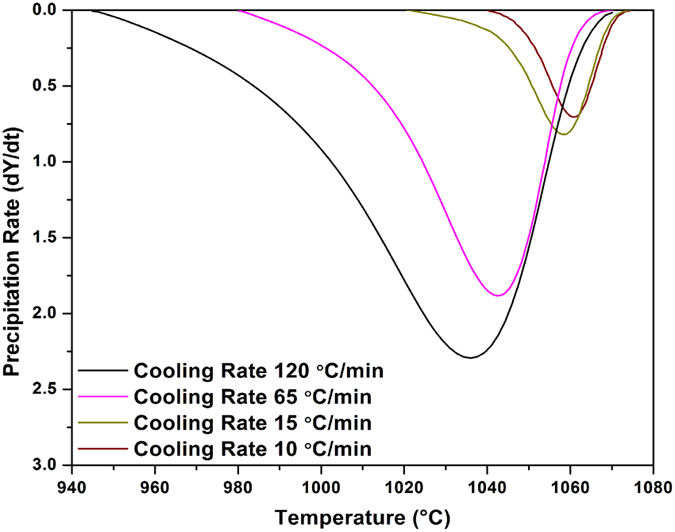
γ′ precipitation rate as a function of temperature for various cooling rates showing the shift of the maxima of the transformation rate curves to lower temperatures with increasing the cooling rate.

Figure 2 displays the evolution of the γ′ precipitated fraction (*Y*) as a function of temperature (*T*) for the four cooling rates. Figure 2 was obtained using Fig. 1(b) and Eqs. (4) and (5). It can be seen that as the cooling rate increases, the curves shift to lower temperatures. For example, for the SC_10_ condition, 25% and 50% of the volume fraction of γ′ particles reprecipitate until 1063 °C and 1059 °C, respectively. However, these fractions are obtained at up to 1040 °C and 1027 °C, respectively, for the FC condition. The evolution of the precipitation rate (*dY*/*dt*) with temperature, calculated from DTA data (Fig. 1(b)), using Eqs. (4) and (6), is shown in Fig. 3 for different cooling rates. An analysis of Fig. 3 indicates that as the cooling rate increases, the maxima of the transformation rate curves shift by 25 °C to lower temperatures, going from 1060 °C for 10 °C/min to 1035 °C for 120 °C/min.

#### Determination of Precipitation Kinetics Parameters

In order to develop a general precipitation equation, the kinetics parameters of precipitation (*n*, *Q*, *k*(*T*)) can be determined from DTA measurements using the non-isothermal JMA model presented in Eqs. (1) to (8), 2, 3, 4, 14, 14, 12, 14. The method used in the present investigation is detailed as follows:

#### Determination of the Avrami coefficient

**Figure 4:**
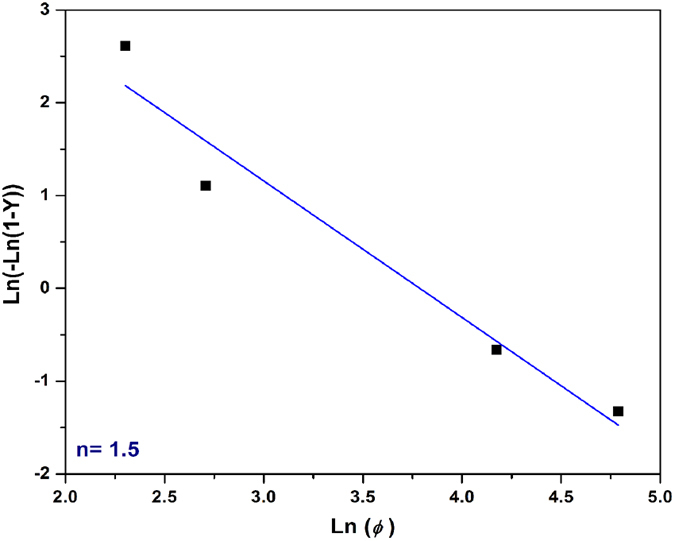
The relationship between 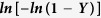 and *Inφ* at 1039 °C for determining Avrami exponent of γ′ precipitation (R^2^ = 0.95).

The coefficient *n* can be determined from the slope of the plot 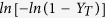 versus 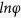. Using the above procedure, the coefficient 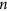 was determined to be in the range 1.5–2.3. In order to avoid displaying similar data, the plot for the minimum value of exponent *n* (*n* = 1.5) is provided only in Fig. 4. It must be noted that in order to obtain reliable data, the transformed fraction, *Y*_*T*_, in Eq. (7) should be considered in the same temperature range for all cooling rates. This temperature range is between 1039 °C and 1070 °C, as shown in Fig. 2.

#### Determination of the Activation Energy

**Figure 5:**
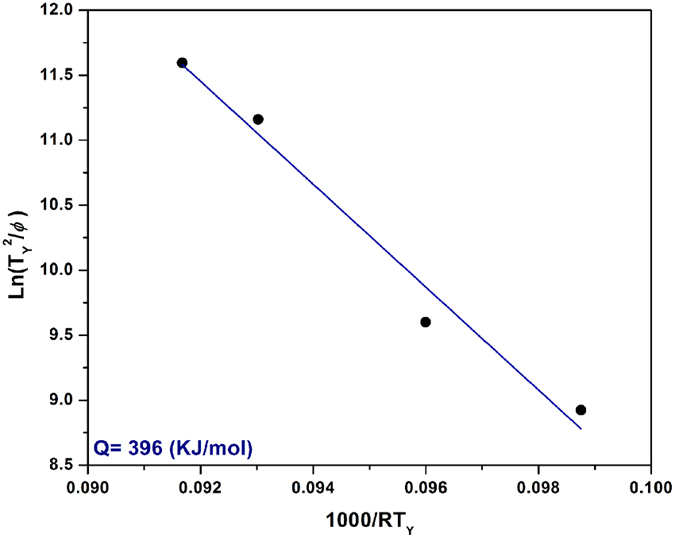
The relationship between 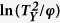 and 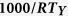 for determining activation energies of γ′ precipitation (R^2^ = 0.98).

Figure 5 shows the plot of 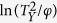 versus 1000/*RT*_*Y*_. Based on Eq. (8) and using Fig. 5, the activation energy for γ′ reprecipitation, *Q*, was obtained as 396 kJ/mol from the slope of the linear fit to the data. The calculated value is close to the activation energy value of γ′ precipitation reported for Waspaloy (398 kJ/mol)^50^. Rosen *et al*.^51^ reported an activation energy of 250 kJ/mol for γ′ precipitation in several wrought Ni-based superalloys. This value for activation energy is close to that of nickel self-diffusion. However, the alloys used by Rosen *et al*.^51^ were of a much simpler composition than the AD730^TM^ alloy used in this study. The smaller number of alloying elements could account for the activation energy being lower than that for AD730^TM^ which has significant amounts of alloying elements.

#### Determination of the Avrami Rate Parameter

The rate parameter *k*(*T*) can be determined from *k*(*T*) = *k*_0_(*T*)exp(−*Q*/*RT*). In order to provide the evolution of *k*_0_(*T*) with temperature, a function needs to be developed. This function should be defined, in which difference between γ′ precipitated fraction (*Y*) calculated using the non-isothermal JMA model and that of the experimental data (Fig. 2) is small. In order to develop this function, MATLAB^®^ was used to optimize the kinetics models for SC_15_ and FC conditions to the experimental values.

Optimization was performed using the Nelder-Mead algorithm as implemented in the MATLAB^®^, and the experimental data inputs used as initial values. The mean squared error, *MSE*, which is used as an indicator of the quality of the model^52^, is the average squared difference between experimental data (Fig. 2) and the calculated values of *Y* at each of the n time steps (*n* = 80–200).


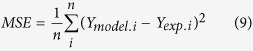


The average difference between the value determined by optimization and the experimental value of the kinetic parameter *k*_0_(*T*) divided by the actual value is defined as the mean absolute percentage error, *MAPE*.


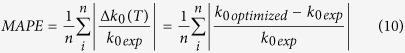


On the basis of the optimization process, *k*(*T*) is developed as a function of temperature as follows:


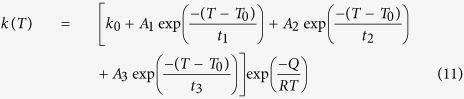


The constants of this function were obtained using experimental data at cooling rates of 15 °C/min (SC_15_) and 120 °C/min (FC). Then, the evolution of the γ′ precipitated fraction (*Y*) as a function of temperature was determined using Eqs. (1) and (3), *k*(*T*), *Q* and *n* at these cooling rates.

**Figure 6:**
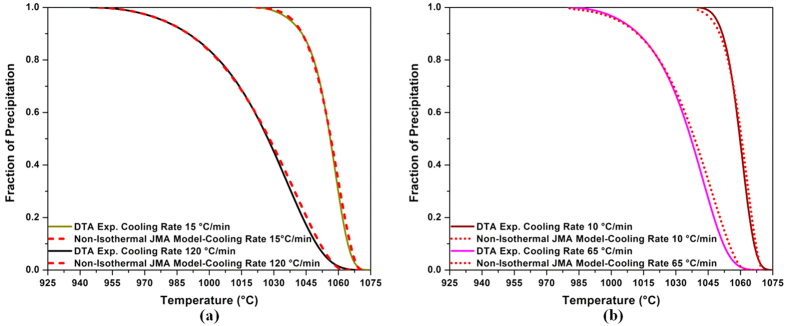
(**a**) Comparison between experimental data of DTA (solid lines) and predictions by the non-isothermal JMA model (dashed lines) using developed k(T) function for SC_15_ and FC conditions (**b**) Validating developed equations of k_SC15_(T) and k_FC_(T) for SC_10_ and IC conditions, respectively. The error between the experimental data (solid lines) and the calculated model (dot lines) is small.

The constants of Eq. (11), 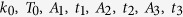, are given in Table 3 for SC_15_ and FC conditions. Figure 6(a) shows the error between the non-isothermal JMA model and the experimental data is negligible. The corresponding differences between input (experimental) values and the optimized value of the kinetic parameter *k*(*T*) are given in Table 4. It can be seen that the model is able to correctly describe the experimental kinetics, as evidenced by small 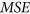 values for SC_15_ and FC conditions.

The developed equations of *k*(*T*) for SC_15_ and FC samples were also validated respectively for the SC_10_ and IC conditions, and the results are shown in Fig. 6(b) and Table 4. The results show that *MSE* and *MAPE* are respectively less than 7 and 6% for the SC_10_ and IC conditions. Therefore, the equation of *k*(*T*) for SC_15_ condition, 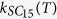, is applicable for slow cooling rates lower than 15 °C/min, and the equation of *k*(*T*) for the FC condition, *k*_*FC*_(*T*), can be applied for cooling rates higher than 65 °C/min.

On the basis of the above calculations, and considering an average value of 1.8 for the coefficient *n*, the kinetics of γ′ reprecipitation in the AD730^TM^ superalloy can be described by the following general equation for both low and high cooling rates:


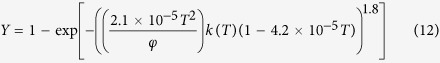


where 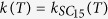 for low cooling rates and 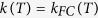 for high cooling rates with the various constants provided in Table 3. It is important to note that the methodology presented in this study can be applied to other Ni-based superalloys where γ′ reprecipitation may take place.

#### Influence of Cooling Rate on γ′ Characteristics

**Figure 7:**
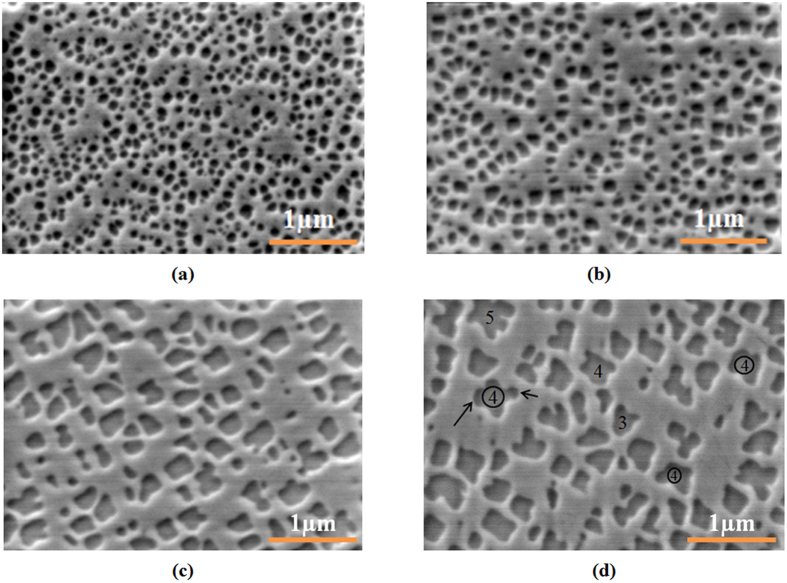
Scanning electron microscopy images of the (**a**) FC (**b**) IC (**c**) SC_15_ (**d**) SC_10_ samples, showing the morphology and size-scale of first generation of γ′ precipitates. Some of protrusions are shown by arrows and their number, *l*, was indicated inside some particle core areas.

Slow, intermediate and fast cooling from supersolvus temperatures result in multimodal distribution of γ′ precipitates, as will be shown later in this section. This multimodal distribution of particles can be divided into different groups based on their morphology and size. Figure 7 shows the morphology and size scale of the first population of γ′ particles for samples cooled under the four investigated cooling rates. The particles’ shapes range from relatively round and regular (Fig. 7(a)) for the FC condition to fully irregular (Fig. 7(d)) for the SC_10_ condition. Papon *et al*.^53^ and Christian *et al*.^54^ suggested that when particle growth is spherical or irregular, the Avrami exponent *n* varies between 1.5 and 2.5. In the present investigation, the *n* value ranges between 1.5 and 2.3, thus indicating that γ′ growth in the AD730^TM^ alloy follows a diffusion-controlled growth process. This is also confirmed by micrographs in Fig. 7.

**Figure 8:**
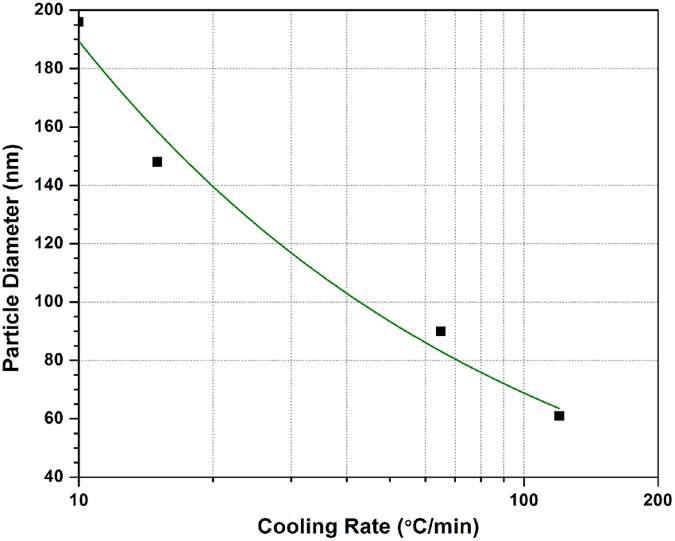
Average diameter of first generation of γ′ precipitate as a function of cooling rate (R^2^ = 0.97).

**Figure 9:**
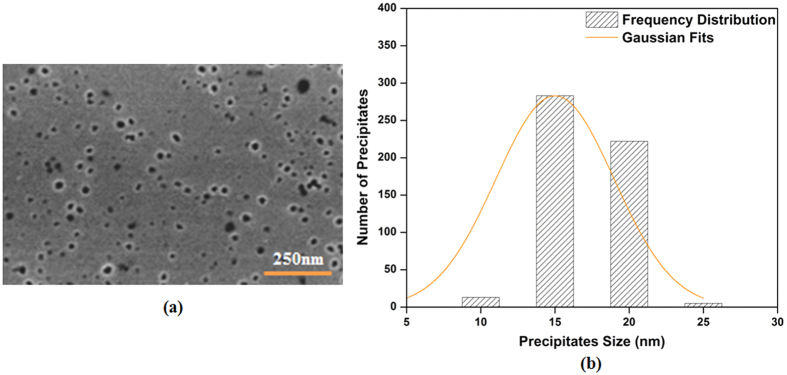
(**a**) Scanning electron microscopy image (**b**) Precipitate size distribution plot of the Gleeble™ 3800 sample.

Figure 8 shows a correlation between average diameters of the first population of γ′ precipitates with the cooling rate. It can be seen that the average sizes of γ′ particles are about 196 nm for the SC_10_ condition and 61 nm for the FC condition. Since γ′ growth during cooling is a diffusion-controlled process^55^, a power law relation could describe the evolution of size with the cooling rate using the data presented in Fig. 8:


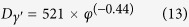


where 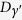 is the precipitate diameter in nm and *φ* is the cooling rate in °C/min. The above relation provides a reasonable prediction (R^2^ = 0.97) of the size evolution over a very broad range of cooling conditions for the first population of cooling γ′ particles. In order to validate the above equations, a heat treatment cycle, 1200 °C/1 minute/ cooling at a rate of 100 °C/sec, was performed using Gleeble™ 3800. The morphology and distribution of γ′ precipitates obtained after the above heat treatment cycle were examined using high magnification SEM micrographs, and are presented in Fig 9(a,b). The results show that a monomodal size distribution of very fine spherical particles, ranging from 7 to 20 nm, with an average size of 13 nm was obtained. The particle diameter should be 11 nm according to Eq. (13), confirming the validity of the proposed equation.

**Figure 10:**
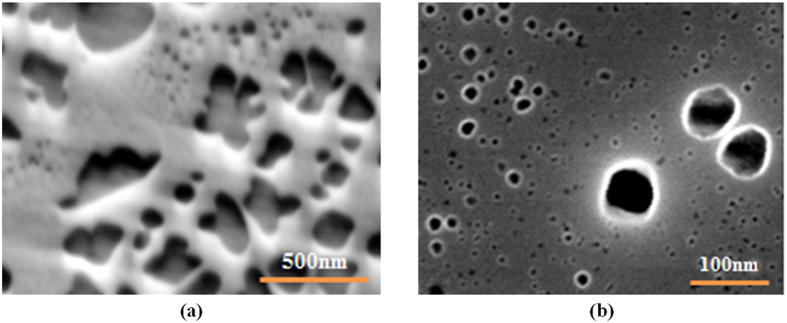
BSE and SE images of the (a) SC_10_ (b) FC samples, respectively, showing first and second population of cooling γ′ precipitates.

**Figure 11:**
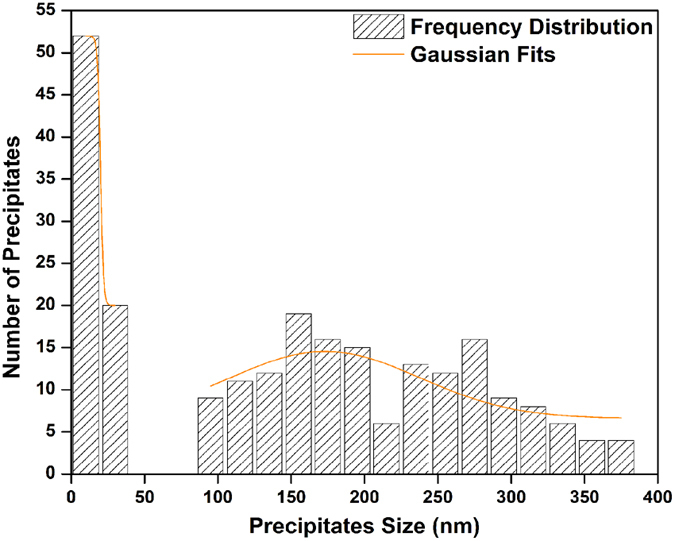
Precipitate size distribution plot of SC_10_ sample showing the size difference between the first and second population of cooling γ′ precipitates.

The second population of cooling γ′ particles for the SC_10_ and FC conditions are shown in Fig. 10(a,b). Particle size measurements indicate that γ′ particles range from 11 to 35 nm for the SC_10_ and from 8 to 12 nm for the FC condition. A detailed particle size distribution analysis carried out over 300 particles clearly reveals a multimodal distribution for the SC_10_ condition as shown in Fig. 11. The size distribution of the third population of γ′ precipitates could not be precisely quantified due to their small size (less than 10 nm). A particle volume fraction analysis showed that the volume fraction of γ′ from the first nucleation burst forms about 85% of the total amount of γ′ precipitates in the AD730^TM^ alloy (which is about 40%) for the SC_10_ condition, indicating that a very small portion of γ′ will be formed as second or third populations of γ′ precipitates.

#### Precipitation during Discontinuous Cooling from Subsolvus Temperature

**Figure 12:**
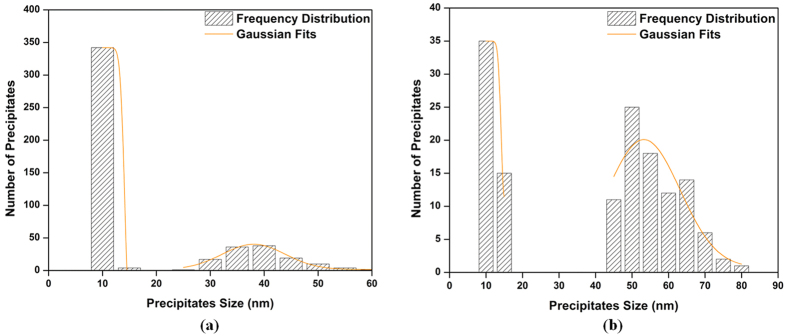
Precipitate size distribution plot of (a) high interrupt temperature (b) low interrupt temperature, from 1100 °C showing the size difference between the first and second population of γ′ precipitates.

Interrupted cooling tests were carried out at a cooling rate of 120 °C/min to study the development of γ′ precipitates during a cooling process which represents interpass cooling during forging. It should be noted that the alloy contains around 7% initial primary γ′ when cooling from subsolvus temperature. The distribution of γ′ precipitates, presented in Fig. 12, reveals a bimodal distribution during cooling from 1100 °C interrupted either at 1040 °C or 780 °C.

**Figure 13:**
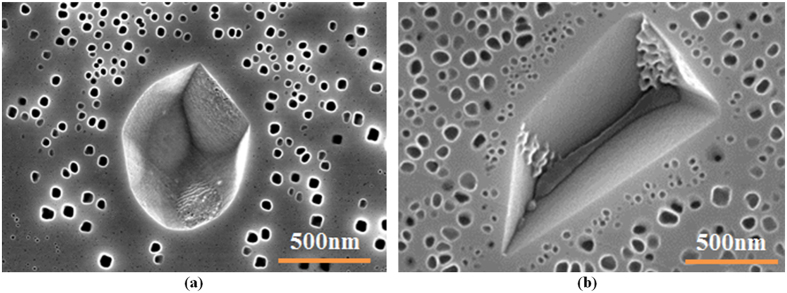
Scanning electron microscopy image of the (a) first burst of nucleation at high interrupt temperature (b) coarsening of first generation of γ′ and second burst of nucleation between initial primary and existing γ′ precipitates at low interrupt temperature. The interrupted cooling consisted of continuous cooling from 1100 °C at a constant rate of 120 °C/min, followed by water quenching at 1040 °C (high interrupt temperature) or 780 °C (low interrupt temperature).

For the interrupt temperature of 1040 °C, the first burst of nucleation occurs in the 1100–1040 °C interval (Fig. 13(a)), and results in the formation of spherical γ′ particles 25 to 50 nm in size and 13% in volume fraction. The second burst of nucleation occurs during water quenching from 1040 °C, and leads to much smaller particles (less than 10 nm), with a small volume fraction of 2.5%. The density of the small particles is about 9 times more than that of the larger precipitates.

For the 780 °C interrupt temperature, the first population of γ′ particles formed in the 1100 °C–780 °C interval was 40 to 80 nm in size, with a volume fraction of about 24%. The average size of these particles is around 60 nm (Fig. 12(b)). This value is very close to the one obtained after continuous cooling (FC), as shown in Fig. 8. This finding indicates that, due probably to low diffusion, very little or no growth of γ′ particles takes place below 780 °C.

Furthermore, as shown in Fig. 13(b), a second burst of nucleation takes place in the 1040–780 °C interval. This second nucleation gives rise to precipitates which are much smaller in size (8 to 15 nm) and volume fraction (3%). The average size and volume fraction for the second population of γ′ particles are the same as those after continuous cooling (FC), 10 nm and 3%. This indicates that a second burst occurs between 1040 and 780 °C in AD730^TM^. This finding is in agreement with DTA results (Fig. 1(a)) which showed that the solvus for the secondary γ′ is around 825 °C for a 120 °C/min heating rate.

## Discussion

### Analysis of DTA Data during the Heating Cycle

The DTA graphs shown in Fig. 1(a) reveal both endothermic and exothermic peaks, indicating that dissolution and precipitation take place during the heating cycle. The peak temperature for precipitation during heating corresponds to the maximum precipitation rate resulting from a competition of two opposing factors: (1) An increase in the diffusivity of the precipitating elements at higher temperatures, which results in an increase in precipitation; (2) A decrease in the driving force for precipitation, due to lower supersaturation, at higher temperatures^33^,^56^. The competition between these two phenomena results in the occurrence of various peaks labeled from A to C in Fig 1(a): A- dissolution of secondary γ′ precipitates; B- precipitation of primary γ′ precipitates; C- dissolution of primary γ′ precipitates. The results obtained are in agreement with those reported by other researchers who observed similar dissolution-reprecipitation sequences in precipitation-hardened aluminum alloys^56^,^57^,^58^. The results also confirm the findings of Boettinger *et al*.^59^, who observed that the limits of the peaks become more and more visible as the heating rate increases.

The full sequence of precipitations of different populations of γ′ particles is only possible when the alloy is maintained below the γ′ solvus temperature of *that* population. For instance, if the alloy is kept at a temperature above the secondary γ′ solvus, but below the primary γ′ solvus (peak B), only primary γ′ could precipitate.

Based on the classical nucleation theory, the critical free energy for the nucleation of a spherical particle (G^*^) is given by^60^:


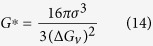


where 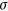 is the interfacial energy between γ and γ′ and Δ*G*_*v*_ is the driving force for precipitation. As shown in Fig. 10, the second population of cooling γ′ particles is very small in size (10 nm) and spherical in shape. Such characteristics result in highly coherent precipitates with the matrix (i.e., minimum interfacial energy and misfit strains), and consequently, very low values for the nucleation barrier G^*^^61^,^62^. DTA results (Fig. 1(a)) show that secondary γ′ particles dissolve at around 785 °C and 825 °C for the SC_10_ and FC conditions, respectively. It has been reported that for each transformation peak in heating, there should be an equal peak for reprecipitation at a few tens of degrees undercooling^59^. Therefore, it is expected that γ′ particles which dissolve during heating will reprecipitate during cooling. This was confirmed in the present investigation by an electron microscopy examination of the microstructure of the samples as shown, for example, in Fig. 10(a,b).

### Effect of Cooling Rate on γ′ Stability during Growth

The morphology of γ′ precipitates is a key factor in determining the properties of superalloys^53^. The irregular growth of precipitates during slow cooling observed in the present work (Fig. 7(d)) is analyzed in the context of the Mullins and Sekerka (MS) model^23^. In the model, the critical particle radius (*r*_*cr*_) for the occurrence of morphological instability, defined by the presence of protrusions on spherical particles, is given by:


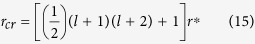


where *l* is the number of protrusions.

The critical radius (r^*^) for nucleation is given by:


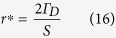


with 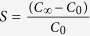 representing supersaturation, and 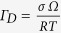 showing capillary constant. *C*_0_ is the equilibrium solute concentration at the precipitate/solid solution interface, *C*_∞_ is the initial solute concentration in the supersaturated matrix, *σ* the matrix-particle interfacial energy, and *Ω* is the increment of precipitate volume per mole of added solute. Finally, *R* is the gas constant, and *T* the absolute temperature.

In order to calculate the precipitate critical radius, an estimation of *Γ*_*D*_ and S must be made. Using *σ* and *Ω* values for superalloys reported by Porter^63^, *Γ*_*D*_ was determined to be 9.7 × 10^−4^ μ*m*. Also, using the Thermo-calc^®^ software, *C*_0_ was calculated for AD730^TM ^33, and consequently, a maximum value of 0.4 was obtained for supersaturation constant ‘S’ when Al and Ti were considered as solutes.

Two-dimensional protrusions on spherical γ′ particles are observed in Fig. 7(d), as indicated by the arrows. The number of protrusions, *l*, was calculated to be 4 for samples slowly cooled from 1200 °C. The measurements were made over 100 particles on 15 different high magnification electron micrographs, such as the one shown in Fig. 7(d).

Using the above data and Eqs (15) and (16), the precipitate critical radius for slow cooling from 1200 °C was calculated to be 78 nm. In order to compare the predicted value with experimental findings, the average core radius of particles was measured and determined to be 89 nm. As for the calculation of *l*, this value represents the average of 100 particles core radii measurements over 15 high magnification SEM micrographs, indicated by circles in Fig. 7(d). A relatively good agreement is observed between measured and calculated values. The difference seen (of about 12%) may be due to the fact that the experimental conditions of Fig. 7(d) do not correspond *exactly* to the onset of instability, as it is very difficult to achieve such precisions experimentally.

**Figure 14:**
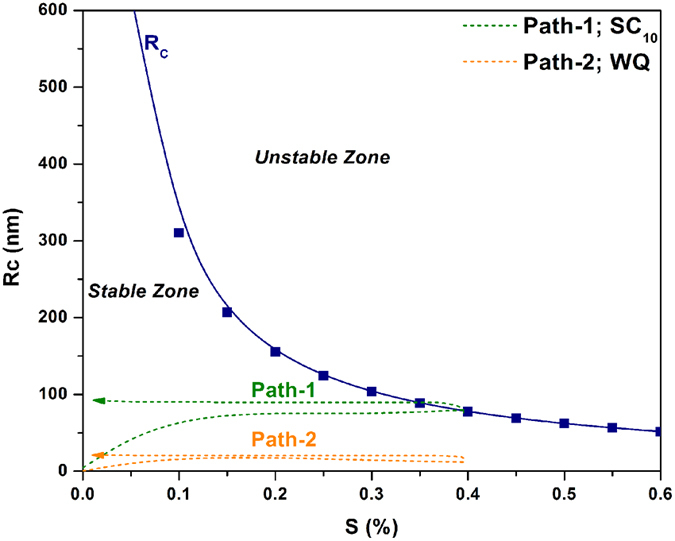
The calculated variation of the critical precipitate radius with supersaturation and schematic illustration of possible growth trajectories of spherical precipitates for SC_10_ (path-1) and WQ (path-2).

The variation of precipitate critical radius versus supersaturation is plotted in Fig. 14 for *l* = 4. It can be observed that precipitates are stable below and unstable above the critical radius 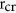. In Fig. 14, the growth routes of precipitates for slow cooling and water quenching conditions are shown schematically by paths 1 and 2, respectively. In both cases, samples were cooled from above the γ′ solvus temperature (*Ts*). According to MS theory^23^, morphological instability depends on the domination of either supersaturation or the capillary effect. Once supersaturation is sufficiently built up, spherical particles become unstable. Therefore, the formation of protrusions during slow cooling (path 1) should occur as sufficient supersaturation can build up during a temperature drop (Fig. 14). Path 1 shows that at the start of nucleation, the shape of the precipitates is mostly spherical due to small supersaturation. As higher supersaturation is present in the case of undercooling, these particles will grow into the unstable zone.

If multiple bursts of precipitates are considered, then after the first burst of nucleation, few γ′ nuclei will form at high temperatures due to the small undercooling below γ′ solvus (as shown in DTA results in Fig. 1(b)). Under these conditions, far from these particles, the γ matrix will remain supersaturated until lower temperatures, i.e., below the peak for the formation of the first population of cooling γ′. Supersaturation will increase further with an additional drop in temperature, such that the second burst of nucleation can occur at these sites (Fig. 10). The nucleation of this population of cooling γ′ will reduce the supersaturation of solute elements, and with the third nucleation burst, most of the supersaturation is consumed, and the γ matrix reaches equilibrium. While it is expected that once the level of supersaturation is decreased, morphological instability should also decrease (return of path 1 to the stable region), γ′ particles keep their morphological instability, as shown in Fig 7(d). This is probably due to the fact that most of the supersaturation is consumed for the second and third nucleation bursts, rather than for the growth of γ′.

In the light of the above analysis, it can be said that the first population of γ′ particles is formed between 1075 °C and 1039 °C, and grow rapidly due to high diffusion at high temperatures, and results in an irregular shape or instability (Figs 2 and 7(d)). The results also indicate that cooling down to room temperature and reduced supersaturation will not necessarily remove any shape irregularity.

In contrast, when high cooling rates are employed (Fig. 14 -path 2), instability is prevented. This is probably due to the high number of reprecipitated fine γ′ particles, which increases in the presence of high cooling rates. Indeed, as shown in Figs 1(b) and 3, higher cooling rates result in increased undercooling below the γ′ solvus temperature, and therefore in higher supersaturation and faster nucleation rates. The proximity of the precipitates results in the overlapping of their diffusion fields and in a rapid reduction of supersaturation around the newly nucleated particles. This overlapping inhibits the instable growth of γ′ precipitates, resulting in the formation of spherical shape particles (Figs 9(a) and 10(b)).

**Figure 15:**
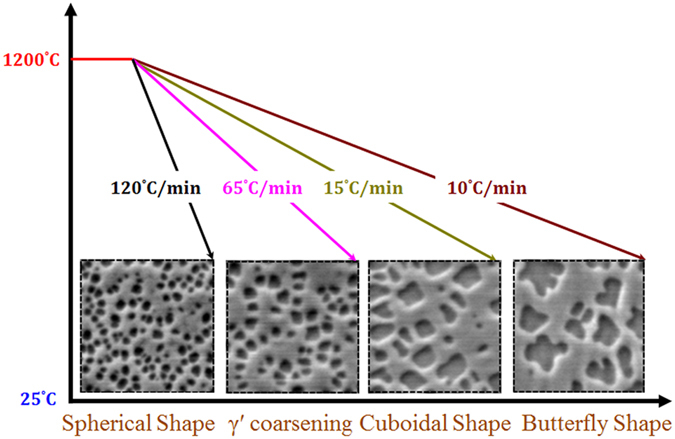
Scanning electron microscopy images showing the morphology evolution of first generation of γ′ precipitates during various cooling rates.

The influence of the cooling rate on the morphology of γ′ precipitates is further illustrated in Fig. 15. It can be seen that during cooling from supersolvus temperature, the first population of γ′ precipitates in AD730^TM^ keeps a spherical shape under fast cooling conditions (FC). The particles coarsen, but still keep their spherical shapes during intermediate cooling (IC). However, with a further decrease in the cooling rate (SC_15_), they coarsen very rapidly, and develop a cuboidal, and then butterfly, shape. In addition, as shown in Fig. 7, the density of the first population of γ′ precipitates is higher for the FC condition, at 35/μm^2^, compared to 3/μm^2^ for the SC_10_ condition. This value for the WQ condition is 85 times higher than that of the SC_10_ condition (Figs 7(d) and 9).

The above analysis shows that supersaturation is not the *only* governing mechanism in the formation of γ′ morphological instability during cooling of the AD730^TM^ alloy and most probably other Ni-based superalloys. Low nucleation density and sufficient diffusivity between precipitates and the matrix are other essential factors for irregular growth in Ni-based superalloys. These findings are in agreement with those of Doherty and Yoo^27^,^64^, who reported that there should be isotropic interfacial energy, low lattice mismatch between the two phases, and a low density of nucleation for the formation of protrusions. γ′ precipitates in Ni-based superalloys normally fulfill all these requirements; specifically, the latter condition was satisfied in this work due to the low nucleation density of the first population of cooling γ′ for the SC_10_ condition.

### Multiple Precipitations during Cooling

As Table 2 and Fig. 1 show, 20 °C and 85 °C undercooling below the γ′ solvus are necessary to enable subsolvus nucleation for slow and fast cooling rates, respectively. According to

Eq. (16), at higher supersaturation, the critical radius for nucleation is decreased, and based on the classic nucleation theory^60^, the nucleation rate will increase. Consequently, the transformation rate will increase gradually from 0 to 0.7 and 2.3%/min respectively for the SC_10_ and FC conditions, as shown in Fig. 3. Thus, for the first burst of γ′ nucleation for the SC_10_ condition, only a limited density of γ′ precipitates will be obtained due to the small undercooling at 20 °C (Fig. 1(b)).

Zener^54^ proposed the following model for the precipitates growth rate:


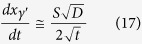


where 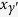 is the precipitate size, *S* the supersaturation and *D* the diffusion coefficient. As the first nuclei are formed and start growing, supersaturation in the matrix is gradually reduced, as does the driving force for nucleation. According to Eq. (17), the growth rate will also be reduced due to a decrease in supersaturation and temperature. Therefore, the transformation rate will decrease and reach 0, and the volume fraction of the particles in the system becomes almost constant. However, as the temperature decreases, the redistribution of the solute elements becomes more and more difficult due to their reduced mobility. This leads to a higher supersaturation level in the region free from the first population of γ′ precipitates, which will then become suitable sites for the second and/or third burst of nucleation (Fig. 10).

For a reprecipitation reaction to occur, the thermodynamics must be favorable (enough driving force), and the kinetics fast enough (small activation energy). Therefore, the transformation rate is proportional to (Kinetic factor) × (Thermodynamic factor). Based on Eqs (14) and (16), the critical radius (r^*^) is high, and the driving force is small for nucleation near equilibrium. Thus, the nucleation rate will be slow for the first population of γ′ due to small supersaturation at slow cooling rates (SC_10_ and SC_15_ conditions). As a result, the reprecipitation process will not be thermodynamically favorable, and most of the transformation will be controlled by growth. At these cooling rates, supersaturation, *S*, is small, and the growth rate is controlled by diffusion coefficient, *D*, according to Eq. (17). The growth of first population of γ′ precipitates, which is a diffusion-controlled process, can occur by replacement of the γ atoms at the γ/γ′ interface with γ′ atoms by normal lattice diffusion involving vacancies. Therefore, the reprecipitation process is kinetically controlled at the SC_10_ and SC_15_ conditions.

As the temperature is lowered during continuous cooling and enough supersaturation and driving force is produced, the probability of nucleation may thus increase rapidly with decreasing temperature, according to Eq. (14).

As Table 2 and Fig. 3 show, in the case of samples cooled at a higher cooling rate (FC), most of the transformation seen will occur at a slightly lower temperature (compared to the SC_10_ condition), where both nucleation and growth rates are higher. For the FC condition, the density of the first population of γ′ precipitates is 12 times, and the transformation rate is 3.5 times higher than that of SC_10_, as shown in Figs 3 and 7. The reprecipitation process of the first population of γ′ precipitates occurs at high temperatures (1075 °C–945 °C) for the FC condition, as shown in Fig. 2. However, a combination of shorter cooling time as compared to the SC_10_ condition, the higher density of particles and therefore overlap of the diffusion fields of precipitates limit the growth rate of γ′ precipitates for the FC condition. This condition will promote a finer size of γ′ precipitates (60 nm) as compared to the SC_10_ condition (196 nm). Thus, the reprecipitation process of the first population of γ′ precipitates is thermodynamically and kinetically favored, and controlled by both nucleation and growth at higher cooling rates (FC).

**Figure 16:**
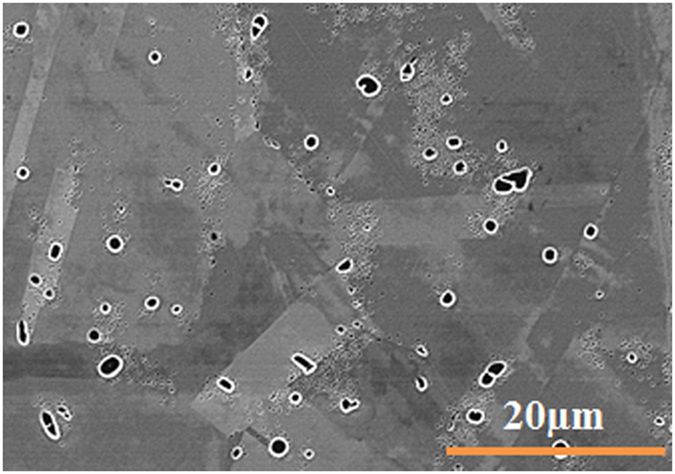
Nucleation of γ′ particles around initial primary γ′ at the γ grain boundaries at high interrupt temperature indicating supersaturation is not uniform at the early stages when cooling from subsolvus temperature.

The experimental results indicate that supersaturation is not uniform at the early stages, when cooling from subsolvus temperature. This is illustrated in Fig. 16, where it can be seen that during cooling from subsolvus temperature of 1100 °C interrupted at 1040 °C, γ′ particles nucleate in the supersaturated matrix around the initial primary γ′ precipitates, which remained undissolved during the solutionizing stage, and mostly lie at the γ grain boundaries.

As shown in Fig. 13(a), for the interrupt temperature of 1040 °C, there is no time for the growth of first generation of precipitates formed during cooling. In addition, the high supersaturation buildup in the matrix during water quenching will be the precursor for the second nucleation burst. Conversely, for the 780 °C interrupt temperature (Fig. 13(b)), existing particles from the first nucleation burst grow by diffusional transfer of atoms toward the precipitates; however, as the temperature is lower, solute diffusivity, and consequently, the growth rate of the precipitates, is slow. Here, the matrix is supersaturated with Al and Ti, and is therefore still in non-equilibrium condition, and thus, a sufficient driving force for a second nucleation burst will be provided at a critical undercooling. This group of precipitates was observed between the initial primary and first groups of precipitates (Fig. 13(b)). It is worth noting that the second group of precipitates is small in size due to the slower movement of solute under these conditions.

## Summary and Conclusions

This study reported the reprecipitation mechanisms and kinetics of γ′ particles in the newly developed AD730^TM^ wrought Ni-based superalloy, with around 40% γ′ particles, but the findings could be also extended to other Ni-based superalloys.

The main conclusions of the present investigation can be summarized as follows:

1. The precipitation of γ′ particles during cooling follows a modified JMA equation, and it was demonstrated that the process is kinetically controlled for the first population of γ′ precipitates at the SC_10_ and SC_15_ conditions. This process is thermodynamically and kinetically favored at the FC condition. The growth parameter (*n*), the activation energy (*Q*), the amount of precipitation (*Y*) and precipitation rate (*dY*/*dt*) were determined for the first time for the AD730^TM^ alloy.
2. A function for the kinetic parameter *k*(*T*) is developed, and reprecipitation kinetics models for low and high cooling rates are proposed to quantify the volume fraction of reprecipitated γ′ particles.
3. High resolution FEG-SEM indicated that with a decreasing cooling rate, γ′ precipitates show morphological instability going from a spherical shape at high cooling rates to butterfly shapes at slow cooling rates.
4. A new equation is proposed on the basis of experimental correlations between the cooling rate and the γ′ precipitate size for continuous cooling from supersolvus temperatures. The proposed equation was validated experimentally for high cooling rates using Gleeble™ 3800.
5. It was found that supersaturation is not the only determining factor in instability formation. Low nucleation density and enough diffusivity between precipitates and matrix are other essential parameters accounting for an irregular growth of γ′ particles in Ni-based superalloys.

## Additional Information

**How to cite this article**: Masoumi, F. *et al*. Kinetics and Mechanisms of γ′ Reprecipitation in a Ni-based Superalloy. *Sci. Rep.* **6**, 28650; doi: 10.1038/srep28650 (2016).

## Figures and Tables

**Table 1:** Chemical composition of AD730^TM^ (wt%)^1^.

Ni	Fe	Co	Cr	Mo	W	Al	Ti	Nb	B	C	Zr
Base	4	8.5	15.7	3.1	2.7	2.25	3.4	1.1	0.01	0.015	0.03

**Table 2:** Values of phase transformations temperatures during cooling for various cooling rates.

Cooling Rate (°C/min)	Temperature of maximum precipitation (°C)	Temperature of precipitation end (°C)
10	1060	1039
15	1058	1020
65	1042	980
120	1035	945

**Table 3:** The constants of k_SC15_(T) and k_FC_(T) equations for SC_15_ and FC conditions, respectively.

Cooling Rate (^°^C/min)	*k*_0_	*T*_0_	*A*_1_	*t*_1_	*A*_2_	*t*_2_	*A*_3_	*t*_3_
15	−1.46 × 10^15^	1293.99	4.5 × 10^15^	1.92	6.09 × 10^15^	22.24	7.45 × 10^15^	22.24
120	−7.07 × 10^15^	1220.01	9.3 × 10^17^	23.62	2.48 × 10^17^	6.57	5.22 × 10^16^	1.4

**Table 4:** The results of optimizing kinetics models to the experimental reprecipitation kinetics for SC_15_ and FC conditions and using optimized k_SC15_(T) and k_FC_(T) models for SC_10_ and IC conditions, respectively.

Cooling Rate: (°C/min)	10	15	65	120
*MAPE*	5.15	1.99	4.1	1.01
*MSE *× 10^4^	6.98	1.86	5.12	0.91

For the optimized parameter k_0_(T), the mean absolute percentage error, MAPE, is given by Eq.(10). Additionally, the quality of the optimization is quantified by the mean squared error, MSE (Eq.(9)).
